# Therapeutic applications of nucleic acid aptamers in microbial infections

**DOI:** 10.1186/s12929-019-0611-0

**Published:** 2020-01-03

**Authors:** Shima Afrasiabi, Maryam Pourhajibagher, Reza Raoofian, Maryam Tabarzad, Abbas Bahador

**Affiliations:** 1 Department of Microbiology, School of Medicine, Tehran University of Medical Sciences, Tehran, Iran; 2 Dental Research Center, Dentistry Research Institute, Tehran University of Medical Sciences, Tehran, Iran; 3 Legal Medicine Research Center, Legal Medicine Organization, Tehran, Iran; 4 Protein Technology Research Center, Shahid Beheshti University of Medical Sciences, Tehran, Iran; 5 Oral Microbiology Laboratory, Department of Microbiology, School of Medicine, Tehran University of Medical Sciences, Tehran, Iran

**Keywords:** Aptamer, SELEX, Single stranded DNA or RNA, Antibiotic resistance, Drug delivery, Biofilm

## Abstract

Today, the treatment of bacterial infections is a major challenge, due to growing rate of multidrug-resistant bacteria, complication of treatment and increased healthcare costs. Moreover, new treatments for bacterial infections are limited. Oligonucleotide aptamers are single stranded DNAs or RNAs with target-selective high-affinity feature, which considered as nucleic acid-based affinity ligands, replacing monoclonal antibodies. The aptamer-based systems have been found to be talented tools in the treatment of microbial infections, regarding their promising anti-biofilm and antimicrobial activities; they can reduce or inhibit the effects of bacterial toxins, and inhibit pathogen invasion to immune cell, as well as they can be used in drug delivery systems. The focus of this review is on the therapeutic applications of aptamers in infections. In this regard, an introduction of infections and related challenges were presented, first. Then, aptamer definition and selection, with a brief history of aptamers development against various pathogens and toxins were reviewed. Diverse strategies of aptamer application in drug delivery, as well as, the effect of aptamers on the immune system, as the main natural agents of human defense against pathogens, were also discussed. Finally, the future trends in clinical applications of this technology were discussed.

## Introduction

Despite significant advances in the prevention and treatment of infectious diseases, expanding day-to-day, however, infections are still one of the leading causes of death, worldwide [1]. During the past few years, the number of just approved antibacterial drugs with a new mechanism of action becomes to decline [2, 3]. Incorrect use of antibiotics leads to side effects, and more importantly, the development of bacterial drug resistance [4]. Due to the increased incidence of antibiotic-resistant bacterial strains, antibiotic selection for infection control is restricted. Antibiotic-resistant bacteria cause numerous death every year and have a direct impact on the economy [5]. Moreover, biofilm-forming bacteria, which result in chronic infections, have developed increased resistance against antibiotic treatments and host defense systems [6]. Therefore, new strategies for infection control and developing of novel antibiotics with less serious side effects, low toxicity and high efficacy seem necessary [7].

The small single-stranded DNAs or RNAs that bind their specific targets with high affinity and selectivity, are called aptamers and produced by a method named systematic evolution of ligands by exponential enrichment (SELEX) or other modified SELEX strategies [8–10]. More than other diverse analytical and clinical applications in diagnosis and treatment, aptamers have also been evaluated as an antimicrobial agent [11]. Today, aptamers are considered in a range of clinical evaluations for a wide range of human disorders. They are powerful and flexible tools for therapeutic goals [12, 13]. In this review, we focused on the role of aptamers in the treatment of microbial infections.

### Aptamer and SELEX

#### Aptamers; definition and history

Aptamers are small single-stranded oligonucleotides (DNAs or RNAs) with low molecular weight (ranging 5–40 kDa), which form a specific three-dimensional structure. Compared with antibodies, aptamers have minor complexity and immunogenicity, in addition to higher affinity and specificity against variety of targets (proteins, cells or small molecules). As well, they have more flexibility and therefore, can attach to interior non-accessible epitopes that cannot be easily targeted by antibodies. They also, show more stability, in addition to the fact that after denaturation, they can return to their proper primary conformation without loss of activity [14]. Several important attributes of aptamers make them a promising alternative to antibodies, including having thermal and chemical stability, simple chemical synthesis that reduce the cost of production and at the same time, it is possible to made modification through chemical synthesis process to make them more adaptable for different applications [15]. siRNAs, fluorophores, radioisotopes, electrochemical systems as well as various nanoparticles can be coupled with aptamers [15, 16]. In addition, aptamers can block the function of target proteins and as a result, they are promising candidates to develop new therapeutics for infections [15].

Aptamers have applied in diverse valuable fields of life sciences, for example, they have been developed as new drug, diagnostic and a bio-imaging agent, drug delivery agent, analytical reagent, food inspection and hazard detection [17]. In addition, aptamers functionalities are diverse, including their ability to block the interaction of receptor–ligand or activating the function of target receptors, as well as they can also act as promising targeting carriers to selectively deliver therapeutic agents to the target cells [18]. However, several disadvantages have been also reported for aptamers. They are sensitive to enzymatic degradation by nucleases in cells and blood circulation. Correspondingly, there are challenges regarding the topical or systemic administration of these drugs [19]. Low molecular weight and short time of aptamers residence in the circulation emerge significant challenges regards to renal filtration control [20]. Some drawbacks can be overcome by appropriated strategies, for example aptamers can be formulated with a large moieties to increase the molecular weight and reduce the renal filtration [21]. The chemical modifications or adding specific functional groups in order to improve aptamers lifetime and their stability in the body, can occur primarily in the SELEX protocol or through post-SELEX modification [22]. One of the challenge resolving strategy is to conjugate the aptamer with nanomaterials that can decrease various of the challenges ahead [19].

#### In vitro *SELEX method*

**Fig. 1:**
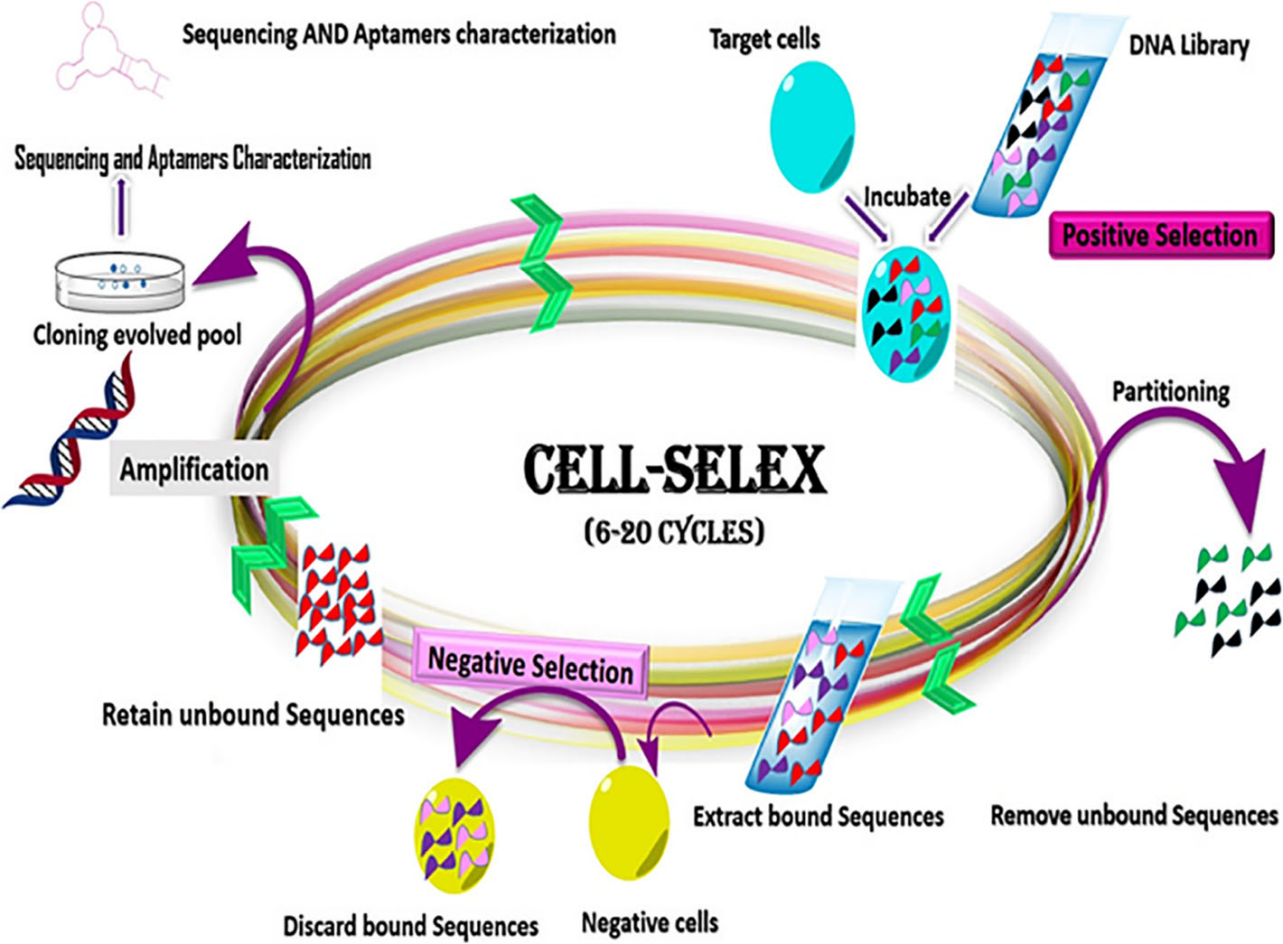
Schematic presentation of DNA aptamer selection using SELEX method

Aptamers are produced through an in vitro procedure, which specifically isolates aptamers for a target of interest and involves repetitive selection-amplification rounds, termed as SELEX [23]. The main advantage of this procedure is to select aptamers, as nucleic acid-based affinity ligands, without any prior knowledge of the target. One of the modified SELEX procedures is the Cell-SELEX, which is used to discover specific aptamers against a whole cell (Fig. 1). In other words, cells are considered as the target in selection process [24]. An oligonucleotide library consists of 10^12^ to 10^16^ distinct sequences. The random region of library is usually 40–100 bases long, flanked by constant primer sequences at both ends, facilitating PCR amplification [25]. The separation of the target-bound sequences from unbound ones via an affinity-based partitioning method and the amplification of the bound sequences by PCR are the important phases of SELEX method. Then, amplified sequences are converted into single strand forms and single stranded oligonucleotides enter the next cycle of SELEX, same as the previous round. The selection cycles are repeated several times to achieve high-affinity sequences in an enriched pool. The identity of DNA pool individuals is determined by cloning and then, sequencing [26] or by next generation sequencing techniques [27]. Several negative selection cycles are developed through a SELEX process to reduce the nonspecific bound oligonucleotides, to enhance the specificity of evolved pool. This type of SELEX has been extensively applied to develop aptamers against bacteria or other pathogenic cells. Low diversity of the DNA library sequence, PCR reagent contamination, contamination of the eluted sequences from the target cells, strong non-specific binding, low frequency of target molecules on the cells and the change in a cellular state can affect negatively the enrichment of the aptamer sequences [28].

#### Anti-bacterial DNA Aptamers

Antimicrobial activities of aptamers have been reported in a number of studies. Some of aptamers targeting bacterial infections are listed in Table 1 and several other important examples were discussed in following paragraphs.

**Table 1:** A summary of aptamers selected against bacterial cells or toxins using SELEX technique, since 2015 till today

Target type	Aptamer Name	Target	Aptamer Type	Sequence of the Aptamer (5′ to 3′)	Kd (nM)	Ref
Bacteria	Lyd-3	*S. pneumoniae*	DNA	TGACGAGCCCAAGTTACCTGCCCCCGAACCATACCACACG ATGCCCCGTACCCCAGCCACCAGAATCTCCGCTGCCTACA	661.8 ± 111.3	[29]
	Apt3	*S. choleraesuis*	DNA	GGCAGGACAACAGCGTGTAGTATCAGCTTACGGTG	41 ± 2	[11]
	BB16-11f	*B. breve*^*a*^	DNA	CTCCCAGGCCGTTGGGGCGTTGCCTGCGT	18.66 ± 1.41	[30]
	CCFM641–5	*B. bifidum*^*b*^	DNA	TGCGTGAGCGGTAGCCCCGTACGACCCACTGTGGTTGGGC	10.69 ± 0.89	[18]
	SAL 26	*S.t*yphimurium	DNA	TAGCTCACTCATTAGGCACATTTGTGGCACCAAATTTGAATTAATCAAGACAGTGTGGTGCATAGTTAAGCCAGCC	123 ± 23	[31]
Bacterial toxin	CT916	cholera toxin	DNA	GGCAAAAAGGATTGCCCAGGTCTGCTGTCTAGCCGGATTC	48.5 ± 0.5	[32]
	S3	SEA	DNA	CCCGCCTCTGAGCATTATTAATGTTATACCTTACGGCTGG	36.93 ± 7.29	[33]
	ML12	LF	DNA	CGAGGGAGACGCGAACCTTCTCGCCTTGGG	11.0 ± 2.7	[34]
	C10	enterotoxin C1	DNA	AGCAGCACAGAGGTCAGATGTATACTTCTAAAATTTGTTTGT ATCTACGATGTTCTTCGTCCTATGCGTGCTACCGTGAA	65.14 ± 11.64	[35]

^a^*Bifidobacterium. Breve* (*B. breve*)

^b^*Bifidobacterium. Bifidum* (*B. bifidum*)

The multidrug-resistant (MDR) strains of bacteria such as *Salmonella* have developed due to inappropriate use of antibiotics. Accordingly, novel anti-microbial agents are required to fight these resistant strains. In a report, the effect of aptamer on colony formation of *S. typhimurium* and *S. enteritidis* was studied that indicated 30 min incubation of specific aptamer with bacteria could result in a significant growth inhibition. The antimicrobial effect of this aptamer might be the result of bacterial cell wall depolarization [36].

*Mycobacterium tuberculosis* (MTB)*,* the bacterium that causes tuberculosis, is another important human pathogen that developed various mechanisms to multiply and survive resistantly in the lungs by confusing the immune system [37]. The polyphosphate kinase (PPK) gene in MTB regulates the intracellular metabolism of inorganic polyphosphate (polyP), which plays a main role in bacterial persistence. The inhibition of PPK activity resulted in the interference of polyP-dependent processes. Aptamer G9 is a therapeutic agent developed against MTB and at the concentration of 1 μM could completely inhibit PPK2 protein of bacteria. Through this process, it exhibited promising antimicrobial activity against MTB [38].

### Aptamer-based therapeutic applications

#### Aptamers in the inhibition of biofilm formation

**Fig. 2:**
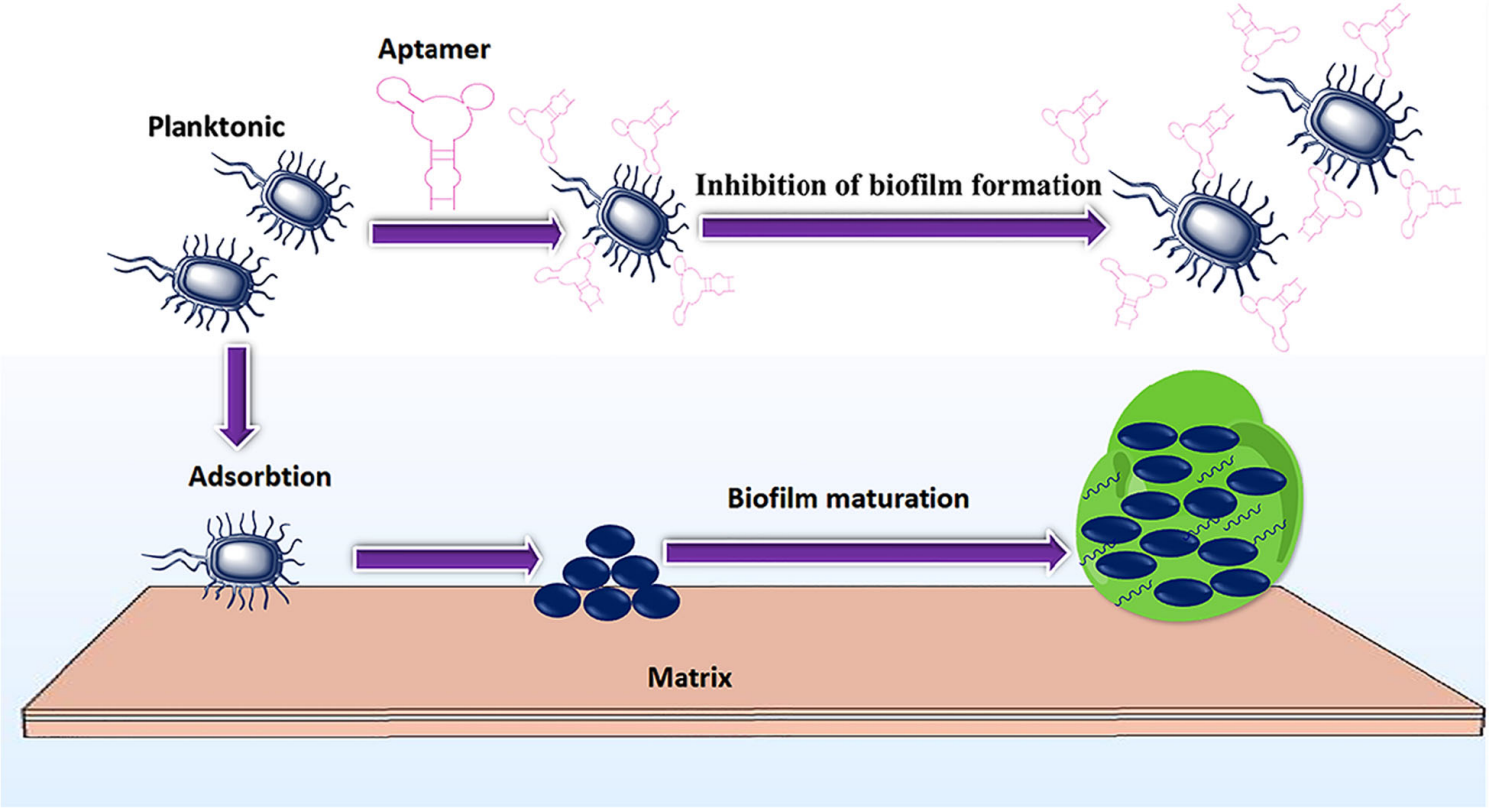
schematic presentation of aptamer effect as an anti-biofilm agent. Aptamer has led to an early stage suppression of biofilm formation. In the absence of aptamer, flagella-mediated motility results in the formation of mature biofilms

Biofilms are persistent communities of microbes that embedded in a matrix of exopolysaccharide and can adhere to surfaces. Biofilms are important in human infections that are hard to treat and cannot be easily eradicated with current antibiotics [39]. Chronic infections by biofilm-forming strains are associated with the accumulation of bacteria, which have a much more complex antibiotic resistance profile, due to the failure of antibiotics in penetrating the polysaccharide layer, as well as, biofilm resistaant against the human immune system [6]. Treatment of biofilm infections is currently a serious problem and antibiotic therapy alone is often inadequate for complete treatment. Biofilms are shown in more than 65% of microbial infections [40]. Motility and initial attachment are believed to be the major features for biofilm formation. It was found that the binding of specific aptamer to the flagella of *Salmonella choleraesuis* could result in the restriction of bacterial rotational frequency, due to the increment of the electrostatic repulsion of cells and surfaces and therefore, preventing the formation of mature biofilms (Fig. 2). In addition, it was shown that in the presence of aptamer, cells were more easily attacked by antibiotics. In this study, it was indicated that the pretreatment of biofilm forming *S. choleraesuis* with aptamer at the concentration of 1.1 μM, could substantially decrease the amount of ampicillin needed for the similar inhibition, as well as, prevented the development of antibiotic resistant strains [41]. It could be concluded that using aptamer pretreatment could eliminate the need for highly potent antibiotics in high dose.

*Streptococcus pneumoniae* is another fundamental pathogen that is involved in the majority of bacterial pneumonias and is the major cause of meningitis, septicemia, otitis media and sinusitis. All wild-type strains are able to form biofilms, in which state they are more invasive for lungs and brain [29, 42]. Among the known DNA aptamers against *S. pneumoniae* (Lyd-1, Lyd-2 and Lyd-3), Lyd-3 aptamer was reported as an effective preventative therapy with anti-biofilm activity, and in combination therapy with appropriate antibiotics, could prevent bacterial colonization. Lyd-3 aptamer at the concentration of 1 μM could reduce the biofilm formation from 100 to 35.8% [29]. The results of this study suggested that specific aptamers, as novel anti-biofilm agents can help to combat the increase rate of antibiotic resistance. Therefore, aptamers can offer a tremendous opportunity for effective treatment of chronic infection, and also, serve a better eradication of biofilm formation bacteria. In general, using aptamers with anti-biofilm activity can lessen the dose and duration of antibiotic therapy in chronic and resistant infections, thus, reduce the risk of abuse and misuse of antibiotic administration [43].

#### Aptamers for the inhibition of microbial toxins

**Fig. 3:**
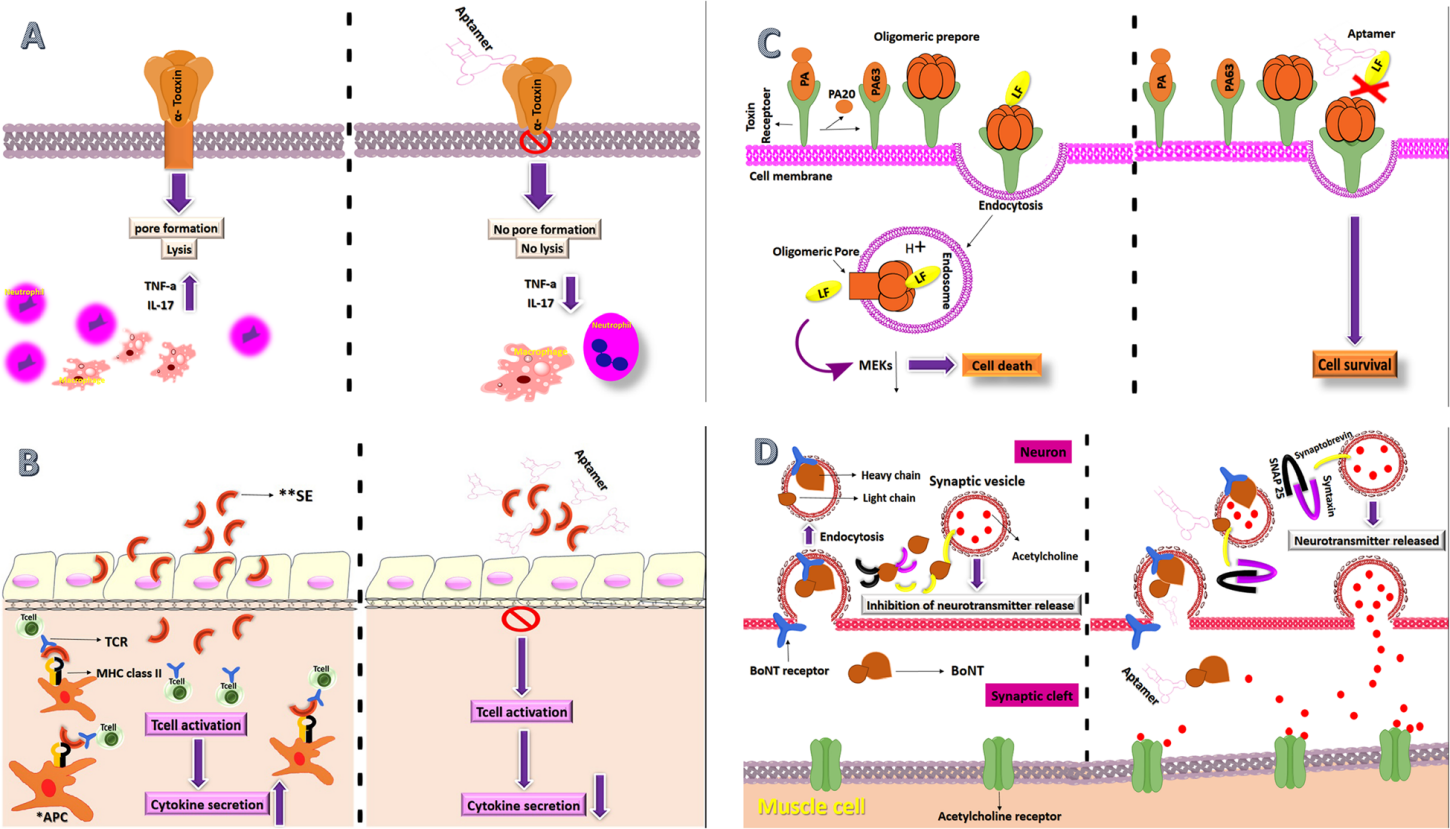
Schematic presentation of aptamer function against different microbial toxins. A) After binding and oligomerization, in the way to create a pore, toxin heptamer inserts into the target cell and leading to cell lysis. In the presence of aptamer, after binding and oligomerization, pore formation does not occur, which inhibit cell lysis. B) *APC: Antigen presenting cell and **SE: Staphylococcal Enterotoxins. SE bound to MHC class II and TCR is expressed on CD^4+^ T cells. MHC class II, SEs and TCR interactions may result in hyper activation of the T cells, leading to the excessive proliferation of T cells and the uncontrolled burst of numerous pro inflammatory cytokines and chemokines. Aptamer inhibits T-cell activation, Therefore, the production of cytokines does not occur. C) PA (protective antigen) binds to the Anthrax Toxin Receptor that then interacts with LF to form the lethal toxin. Translocation of LF through the PA heptamer channel into the host cell cytosol results in cell death. Aptamers block the active site of LF and lead to cell survival. D) BONT (botulinum) is internalized into endosomes. In cytosol, proteolysis by the light chain cleavages SNARE proteins (synaptobrevin, SNAP25 and syntaxin) in the neurons and prevents release of the acetylcholine. Aptamers were bounded to light chain of toxin and caused a strong inhibition of endopeptidase activity

Toxins are powerful bioactive molecules that are produced by various bacterial pathogens and play a key role in the pathogenesis of bacteria. They are essential virulence factors allowing the organism to harm the host, through various physio-pathological pathways. Some toxins may target macrophages and neutrophils, resulting in the reduction of intrinsic immune responses and as a result, provide a suitable environment for active bacterial proliferation [44]. Bacterial toxins may degrade signaling pathways and disrupt the structure of host cells and lead to the persistence of infection [45]. Aptamers have been demonstrated as powerful inhibitory agents against bacterial toxins (Fig. 3).

*Staphylococcus aureus* has been the most important human pathogen among the staphylococci with virulent toxin, by far. Although this organism is frequently a part of the normal human microflora, it can cause important opportunistic infections under the suitable conditions. *S. aureus* may cause a wide range of infections and quickly becomes resistant against many antibiotics [46]. One of the key virulence factors of *S. aureus* is alpha toxin, which can form heptameric pores in target cell membranes and cause tissue damage [46, 47]. Overexpression of TNF-α shows a major role in the destruction of epithelial cells and cell death, which caused by *S. aureus* alpha-toxin [48]. Four aptamers, AT-27, AT-33, AT-36 and AT-49 were developed, which specifically prevented the alpha toxin-induced cytolysis and activation of TNF-α and IL-17. The viability of cells in the presence of toxin after treatment with these four aptamers increased from 50 to 60% to 85–90% [47]. Moreover, enterotoxin B (SEB) from *S. aureus* can cause staphylococcal toxic shock syndrome (TSS), which is rarely observed with other enterotoxins types. SEB, one of the virulence agents of *S. aureus*, acts as superantigen, and stimulates T cells to produce a storm of cytokines, which manifested to clinical complications and food poisoning [49]. A11 is a reported aptamer that prevented SEB-mediated expression of inflammatory cytokines genes, which mediating septic shock. A11 could significantly inhibit SEB activity at the onset of the toxicity cascade, prior to pathological activation of T cells [50].

Another example is S3 aptamer, which could bind to *Staphylococcal* Enterotoxin A (SEA), as the main component of *S. aureus* pathogenesis. It effectively neutralized SEA, as well as, significantly decreased cytokine secretion. The blockage of the toxic cascade, caused by SEA, may be an effective approach for the treatment of SEA-related illnesses [33].

*Bacillus anthracis* is another important pathogen of the genus *Bacillus* that causes the emergence of anthrax. The virulence of *B. anthracis* depends on the production of anthrax toxin. The anthrax toxins consist of three components. One of them is protective antigen (PA), and the active PA heptamer can bind one or more molecules of edema factor (EF), lethal factor (LF) or both that facilitates the pass of this complex into the cell. EF with PA forms edema toxin. A main virulence factor of *B. anthracis* is the LF with zinc metalloprotease activity and is capable of cleaving a *kinase* enzyme, known as mitogen-activated protein kinase (MEK) family. Blocking the interaction between LF and MEK1 leads to the reduction of LF toxicity. An ssDNA aptamer (ML12) was reported that could bind strongly to and was an impressive inhibitor of LF. ML12 by blocking the active site of LF, prevented the protease activity and served as a promising drug candidate for neutralization of LF toxicity [34].

Another important toxin related syndrome is Botulism, caused by *Clostridium botulinum*. Botulinum neurotoxins (BoNT) are one of the most lethal toxins for humans, which also considered in biological weapons. Types A, B and E are the main causes of human illness and type A is the strongest one among other serotypes. This toxin is a 150 kDa protein, which is cleaved into 100 kDa and 50 kDa proteins linked by a disulfide bond; light chains (LCs) with zinc-endopeptidase activity and a nontoxic subunit (heavy chain). The carboxyl-terminal of the botulinum heavy chain binds to the target nerve cells and stimulates endocytosis of the toxin molecule. The LCs inactivates the proteins that regulate the release of acetylcholine, and results in the blocking of neurotransmission at peripheral cholinergic synapses. Three aptamers for BoNT were reported that were bounded to LC with inhibition constants in the low nM range and could cause a strong inhibition of endopeptidase activity [51].

#### Aptamers as intracellular delivery vehicles

Conventional therapy with antibiotics had faced serious restrictions, including low intracellular drug concentrations, loss of the drug through efflux pumps or enzymatic degradation and development of multi-drug resistance (MDR) strains. Drug delivery systems have introduced numerous profits, including improving treatment by increasing the efficacy and duration of drug activity, increasing patient satisfaction by reducing the required dose of drug, adaptation with convenient routes of administration, delivering to the target site to reduce the unwanted effects, and the reduction in cost of treatment that are generally due to their good stability, expanded loading capacity and improved physicochemical features [52].

**Fig. 4:**
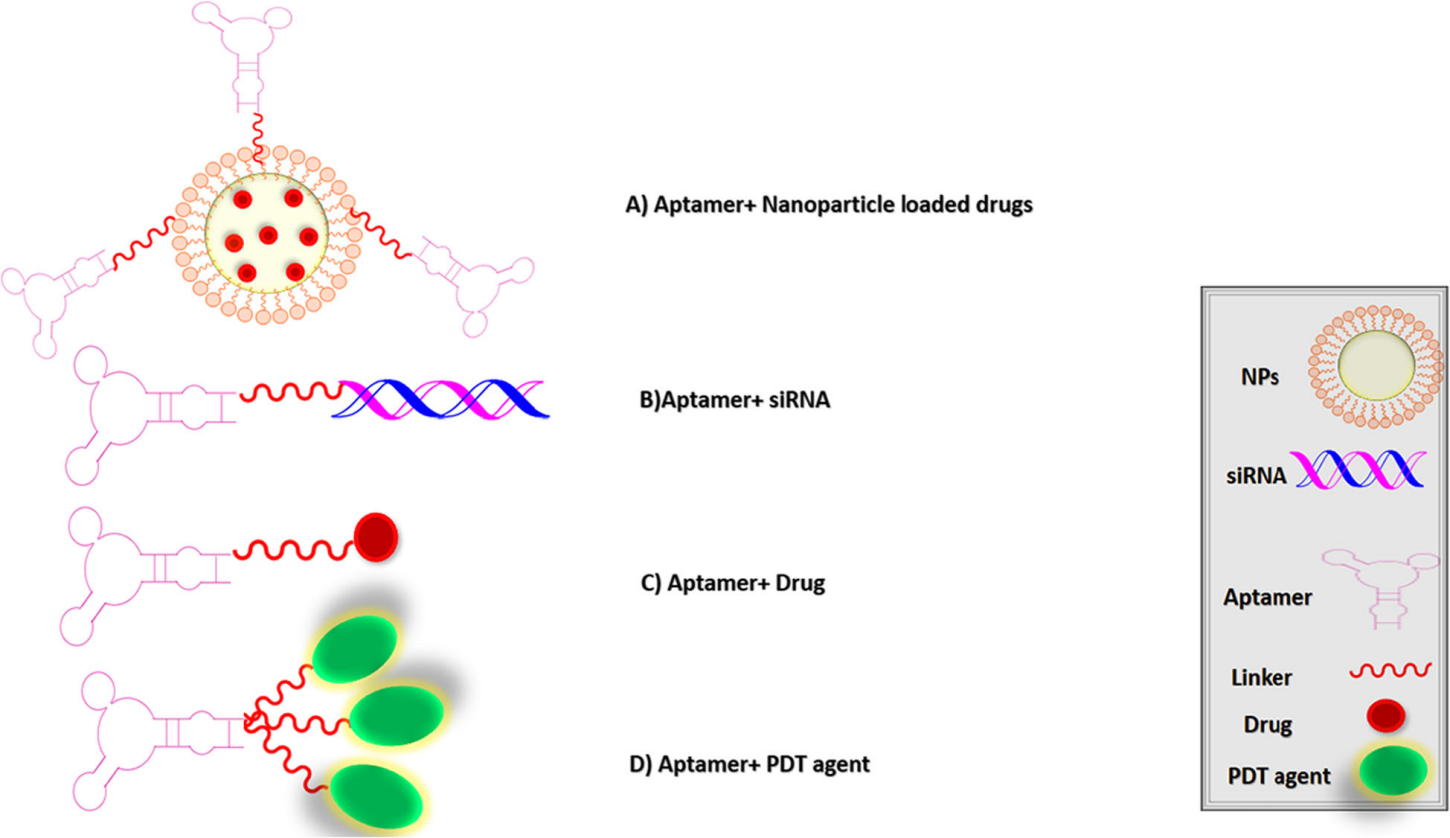
Schematic presentation of the therapeutic applications of aptamers for microbial infections

Aptamers can bind specifically to cell surface receptor molecules. Therefore, aptamer-based delivery systems can improve the cellular entrance of therapeutic agents. Aptamers can be designed for a wide range of applications through coupling with nanoparticles, drugs or other nucleic acids (nano carrier/ drug carrier) and play a major role in cell internalization (Fig. 4) [53].

#### Aptamer-nanoparticle (NPs) conjugates

NPs are small particles with the size of 1–100 nm, which exhibited the potential of antimicrobial activities. Thus, they are appropriately suited to fight microbial infections [54]. Antibiotics encapsulated in NPs have a large capacity to be replaced with antibiotics in free form. There are numerous benefits in nano-encapsulation strategy such as very-small size, a big surface-zone-to-mass ratio, high loading capacity and high reactivity, protection of antibiotics against physical inactivation, improvement in antibiotics pharmacokinetics, facilitation of the antibiotic release in infection area and reducing the required dose of drug [55, 56]. The assembly of aptamers- nanoparticles can result in an increased targeting and more efficient therapeutics [57]. Most NPs possess acceptable biocompatibility, and they can defend nucleic acids from the destruction caused by nuclease digestion. In this regard, aptamer conjugated NPs are promising tools in drug delivery [58].

Antimicrobial peptides (AMPs) are considered as a suitable drug candidate to treat MDR bacteria, but they are unstable in vivo and have a short half-life with low penetration into mammalian cells [59]. HPA3P^His^, as an antimicrobial peptide, loaded onto a gold nanoparticle conjugated with *Vibrio vulnificus* specific DNA aptamer. The resulted composite promoted bacterial degradation by destroying the integrity of the *V. vulnificus* membrane, thus, caused bacterial cell death. In this study, following intravenous injection, AuNP-Apt^His^-HPA3P^His^ induced 0% mortality in infected mice, compared to the HPA3P^His^ alone (100% mortality). AuNP-Apt^His^ exhibited bactericidal activity in concentrations more than 5 nM. AuNP-Apt^His^-HPA3P^His^ had several advantages; 1) The AuNP-AptHis enabled the efficient intracellular delivery of HPA3P^His^ to the host; 2) It increased the stability of HPA3P^His^ by protecting it from proteolysis; 3) It helped avoiding short-term reiterative administrations and reducing overall cost of therapy; 4) This system was simple and did not show any evident host toxicity [60]. On similar note, Yeom, J.-H et al. reported a C-terminally hexa-histidine-tagged A3-APO (A3-APO^His^) AMP, which were loaded onto AuNPs conjugated with His-tag DNA aptamer (AuNP-Apt^His^) in order to successfully and effectively deliver the antimicrobial peptide into *Salmonella*-infected HeLa cells. Anti- His-tag aptamer improved the attachment of His-tag-AMP to NPs. The system was designed to improve AMP stability and its intracellular delivery and had potential impact in the elimination of intracellular *Salmonella typhimurium* infection by disrupting the bacterial cell. In vivo evaluation of this system in mice also indicated promising results [59].

The effect of aptamer-conjugated nanoparticles on the biofilm formation has been also evaluated. In a report, single-walled carbon nanotubes (SWNTs) with significant antimicrobial activity were utilized as a nanomaterial to detect *Pseudomonas aeruginosa* as well as it could increase the toxic effect of antimicrobial agents on bacterial biofilms. A specific aptamer (PA-ap1) against *P. aeruginosa* with the binding affinity in nM range had been developed. The conjugation of PA-ap1 to SWNTs could produce an effective concentration of the particles around bacterial cells, resulting in the disruption of bacteria accumulation. Compared with PA-ap1 and SWNTs alone, PA-ap1-conjugated SWNTs, caused a 36-fold higher inhibition of the biofilm formation. So, PA-ap1 increased the efficiency of anti-biofilm activity of SWNT [61].

Another effective aptamer- nanoparticle complex against biofilm was also reported. Graphene oxide (GO), is an extensively used chemically modified graphene derivative, which has anti-microbial activity. A research group was studied the conjugation of four different aptamer sequences (ST-1, ST-2, ST-3, ST-4) with GO in order to apply as an anti-biofilm agent in *S. typhimurium* model. ST-1 aptamer -conjugated graphene oxide (ST-1-GO) provides a powerful platform to impede biofilm formation through a targeted manner, showing the synergistic effect of aptamer and GO against biofilm formation. By using ST-1-GO, effective anti-biofilm concentration of GO was decreased from 125 mg/L to 100 mg/L, while the anti-biofilm effect was enhanced from 66.4 to 71.4%. Actually, GO entry into the biofilm was facilitated by aptamer ST-3 and they could reduce the cellular membrane potential to inhibit bacterial growth at the same time. The results support that ST-1 aptamer can be a therapeutic candidate for drug delivery [62].

#### Aptamer-siRNA/miRNA conjugates

Short interfering RNA (siRNA) are 21–23 base pairs (bp) in length and as an exogenous agent can experimentally manipulate gene expression. siRNAs, compared to similar therapeutic agents, exhibited minimal toxicity and high stability. siRNAs had demonstrated to have a promising modulatory effect on virulence, drug resistance and pathogenesis [63]. The use of siRNA–aptamer conjugates for drug delivery can result in the increase of favorable therapeutic efficacy and safety [24].

During a viral challenge, infected cells encoding miRNAs is capable of influencing the host cell RNAi system and induce transcriptional gene silencing (TGS) at the viral genome, thus forming latency in viral infection. In addition, the presentation of exogenous siRNA, miRNA and shRNA into infected cells that target integrated viral promoters can significantly repress viral transcription through TGS [64]. In HIV-1-infected cells, gp120 aptamer -LTR-362 siRNA conjugates induced TGS with a ~ 10-fold suppression of viral p24 levels and also, reduced CD4^+^ T cell depletion. Actually, gp120 aptamer functionally delivered LTR-362 siRNA and induced TGS of HIV-1. Generally, receptor-directed aptamers can deliver small RNAs that functionally modulate HIV-1 infection as well as viral transcription [65].

#### Aptamer- drug conjugates

According to the presence, as well as easily add functional groups to the terminals of oligonucleotide sequences, drug molecules can be easily bound to aptamers [66]. For example, ampicillin, by itself, is ineffective against biofilm formation in *S. choleraesuis* infections. Aptamer - ampicillin conjugate could produce synergistic results against biofilms. Aptamer as the antibiotic carrier, helped ampicillin to penetrate into the biofilm and kill the bacteria or reduce biofilms’ tolerance to drugs [11].

Tetracycline-loaded hydrogels were considered for controlled release by using DNA oligonucleotide as aptamer similar agent, that increased tetracycline loading and sustained its release. The study was designed based on the physicochemical features of nucleic acid sequences and possible interaction between oligonucleotide and tetracycline. This oligonucleotide could rise the partition coefficient of tetracycline between hydrogels and drug loading solutions and also, it could slow the release of tetracycline from hydrogels to increase bacterial inhibition [67].

#### Aptamer conjugates for photodynamic therapy (PDT)

PDT is a non-invasive technique includes three items: visible light, a photosensitizer (PS) and oxygen and has approved as an antimicrobial therapy in the treatment of infections [68, 69]. The studies discussed on the applications of aptamer-conjugated to a PS indicated that this complex could increase the selectivity of PDT [70]. Photothermal therapy (PTT) belongs to the group of phototherapy that produces reactive oxygen species, thereby inducing oxidative stress. They are slightly invasive by low toxicity for the nearby healthy tissue, in which photon energy is converted into heat [71, 72].

Methicillin resistant *S. aureus* (MRSA) is a famous pathogen in producing serious infections that resistant to many of the common antibiotics. In order to increase the MRSA treatment efficiency, an aptamer-functionalized gold nanorods (Apt@Au NRs) was introduced as a targeted PTT. It specifically bound to MRSA cells, resulting in recognition and inactivation of MRSA cells through hyper-thermia. The immobilization of aptamer on the surface of AuNRs led to the increase in targeted MRSA cell- binding affinity [73]. Light strongly is absorbed into the gold nanoparticles and causes the quick conversion of photon energy into heat [71].

### Aptamer as an inhibitor of immune cells’ invasion

**Fig. 5:**
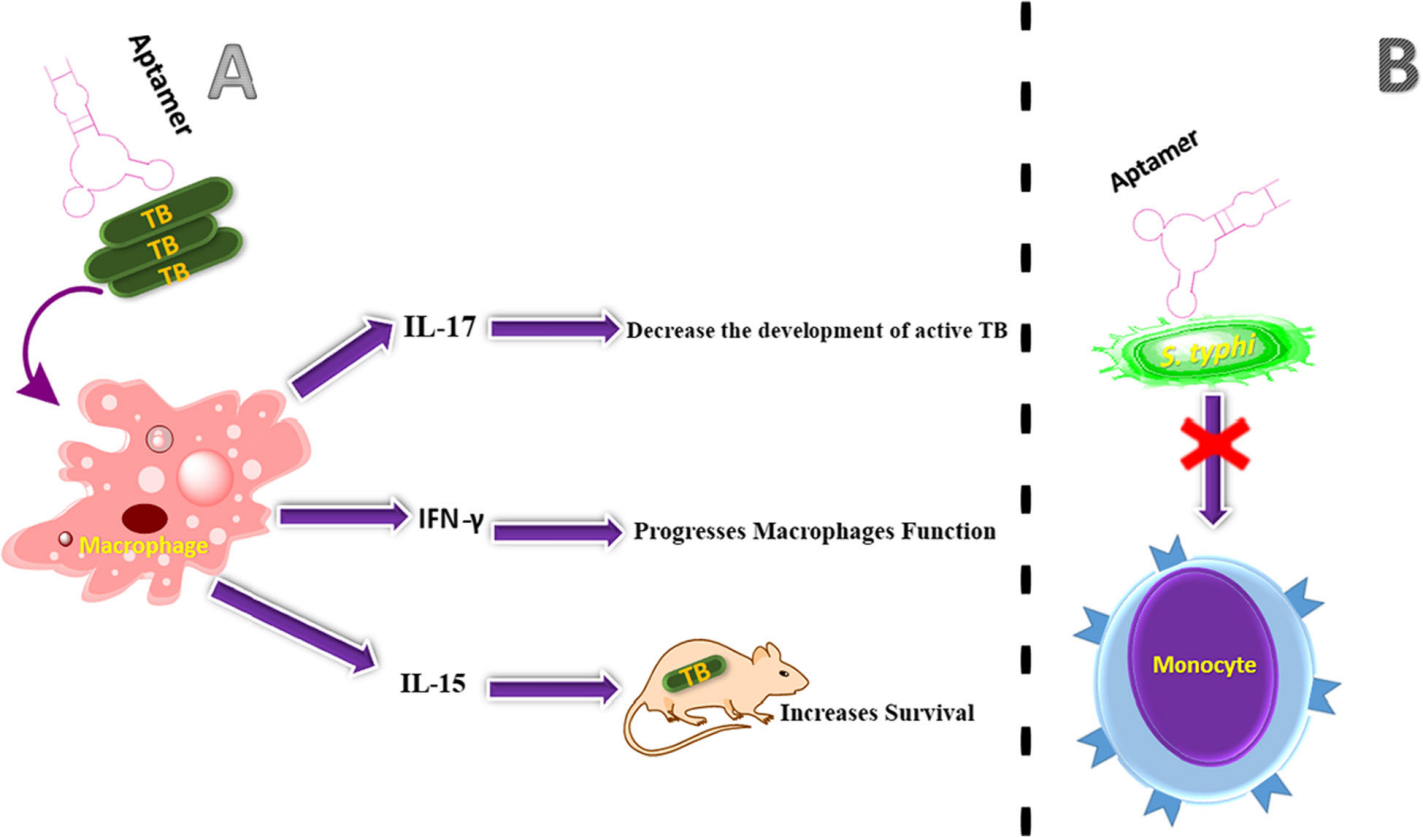
Schematic presentation of aptamer effect as an inhibitor of immune cell invasion A) The aptamer binding to MTB leads to the inhibition of virulent MTB invasion to macrophages. B) The aptamer with *S.typhi* leads to the entrance inhibition of bacteria into human monocytic cells.

Bacterial specific aptamers can be a promising agent in the prevention of pathogen invasion to immune cell (Fig. 5). Some of the examples were illustrated in following section.

#### Inhibits the invasion to macrophage

Some of infectious pathogens invade the host immune cells, which made the treatment complex. For example, during the past 200 years, there are almost 1 billion deaths caused by MTB (an intracellular bacterium), which survives and multiply within macrophages. Most of the macrophages are killed by the bacilli that makes it harder to control infection in MTB-HIV coinfection and in the cases of appearance of multiple drug resistant strains [74, 75]. After the activation of macrophages, MTB begins to multiply within cells, then, other macrophages have developed an enhanced ability to kill the bacilli infected cells. Since, invasion to the macrophages is a major step of the MTB infection process, specific aptamers against virulent markers of MTB can prevent its invasion to the immune cell. NK2 aptamer as MTB specific DNA aptamer, with a Kd in the nM range, considerably inhibited virulent MTB invasion to macrophage, in vitro*,* and virulent MTB that was affected by aptamer could stimulate *IFN-γ*, IL-15 and IL-17 secretion from macrophage [76]. *IFN-γ* led to enhance macrophages function against MTB. IL-15 and IL-17 increased survival of MTB - infected mice and decreased the development of active MTB, respectively [77–79].

#### Inhibits the invasion to monocytic cell

*Salmonella enterica* serovar *typhi* (*S. typhi*) produces a feverish disease called typhoid fever. IVB pili is expressed by *S. typhi*, which is the main character in the development of epidemics of typhoid fever. Monocytes and macrophages are critical effector molecules that play a crucial role in defense against *Salmonella* bacterial infections. Several aptamers were recently developed that specifically bind to type IVB pili and can interfere with bacterial pathogenesis, such as inhibit the bacterial aggregation or inhibit the entry of bacteria into human monocytic cells. In this study, RNA aptamers generated based on SELEX procedure, which one of them, S-PS8.4, exhibited the binding affinity in the nM range for *S. typhi* type IVB pili [80].

### Application of Aptamers in the treatment of viral infections

Viral infections are a threat to human health. Effective and early treatment can improve the prognosis of the disease. Due to repeated virus mutations and viruses escaping the immune system, many viral drugs are ineffective. In addition, many antiviral drugs can cause severe side effects or may interfere with other drug agents and lead to low efficacy. As a targeted treatment, aptamers can be applied against a variety of viruses. They can be effective in antiviral therapy through several methods [81]. They can suppress viral nucleic acid replication by inhibiting nucleocapsid assembly that reduces extracellular DNA. Apt.No.28 against hepatitis B virus (HBV) exhibited this feature [82]. Aptamers can impede virus binding to host cells. As an example, the influenza virus hemagglutinin binds to the sialic acid receptor of the host cell, playing a fundamental role in initiating of viral infections. A DNA aptamer, A22, obstructed influenza virus infection by blocking the virus attachment to host cells [83]. Also, T14 and F34 aptamers prevent rabies virus from entering the host cells by blocking the interaction between virus and host cell receptors [84]. Moreover, in the penetration process of herpes simplex virus, the gD protein is a major determinant of herpes simplex virus-1 (HSV) entry. Two aptamers were selected, which interfered the interaction of the gD protein and the HSV-1 target cell receptor [85].

In fact, various enzymes are important in the virus replication cycle. Enzymes are attractive targets that can be targeted by antiviral aptamers [81]. RNA aptamers have been selected which could inhibit the enzyme activity of HIV tat and reverse transcriptase [86], hepatitis C virus (HCV) NS3 protease/helicase [87], NS5B RNA-dependent RNA polymerase [88], SARS (severe acute respiratory syndrome) coronavirus (SCV) NTPase/ Helicase [nsP10] [89]. Some aptamers developed as antiviral therapeutic agents are summarized in Table 2.

**Table 2:** A summary of several aptamers developed for the treatment of viral infections by SELEX method

Organism	Type of aptamer	Target	Aptamer therapeutic effect	Ref
HBV	DNA	Core protein	-Prevent the assembly of the nucleocapsid -function by suppressing HBV replication	[82]
HCV	DNA	Envelope Protein	-Inhibition of virus binding to host cells	[90]
Influenza A virus	DNA	Hemagglutinin protein	-Blocking the binding of virus to target cell receptors -prevention of the virus invasion into the host cells	[83]
Ebola Virus	RNA	VP35	-Disrupt the eVP35− NP interaction	[91]
HIV-1	RNA	gag protein	-Inhibit HIV-1 replication	[92]
HSV-1	RNA	gD protein	-Blocking the gD functions	[85]
Vaccinia virus	DNA	Hemagglutinin protein	-Recognize proteins expressed on the surface of VV- infected cells	[93]
Influenza B Virus	RNA	Hemagglutinin protein	-Inhibited HA-mediated membrane fusion	[94]
SCV	RNA	nsP10	-Inhibit double-stranded DNA unwinding activity of the helicase	[89]
Rabies virus	DNA	Blocking the interaction between rabies virus and permissive host cell receptors	-Reduced viral replication in an in vitro infection model	[84]

*VP35* viral protein 35, *NP* nucleoprotein, *nsP10* NTPase/Helicase

Moreover, aptamers may be applicable in drug development. One example in this regard is RNA aptamers developed against aminoglycoside by in vitro selection to discover the interactions between aminoglycoside and natural RNAs, including HIV RNAs, which may have similar mechanism in their interactions. The in vitro selected aminoglycoside aptamers seem to have valuable applications in the discovery of the pathogenesis and treatment of HIV infection, including molecular mechanism of interaction with aminoglycoside antibiotics, and screening of efficient anti-HIV drug candidates [95].

#### Application of Aptamers in the treatment of parasitic infections

Parasitic infections having a serious impact on the health, worldwide [96]. Many of the drugs used today are not very effective and some of them can cause severe complications, so trying to find novel agents to control parasitic infections has great attention [97]. There are various strategies on the development of parasite-specific aptamers. They can prevent the interaction between the parasite and the host*. Trypanosoma cruzi* is a parasite that invade host cell. Ulrich, et al. identified an aptamer that could block receptor-ligand interactions between *T. cruzi* and epithelial monkey kidney LLC-MK2 host cells and thus, inhibit cell invasion [98]. Aptamers can inhibit the function of proteins [83]. Erythrocyte membrane protein 1 (EMP1) is a key factor in the pathogenicity of the *Plasmodium falciparum*. This protein involved in parasite adhesion to erythrocytes. Specific RNA aptamers are able to detect this protein on the surface of infected erythrocytes. So the aptamers act as an anti-rosetting drug [99]. Other aptamers that target protein are AptLiH2A#1 and AptLiH2A#2 that distinguished the LiH2A protein. These ssDNA aptamers bind to *Leishmania infantum* H2A with high affinity and does not identify other *Leishmania* proteins [100]. Another example for the inhibition of the function of proteins are poly (A)-binding protein (PABP) DNA aptamers (ApPABP#3, ApPABP#7 and ApPABP#11). They were able to bind PABP that is an essential protein for *leishmania infantum* survival. ApPABP#11, degrades the binding of PABP to poly(A), this feature may be applied in regulating the function of PABP in vivo [101]. EhCFIm25 is critical for parasite virulence and survival, EhCFIm25 silencing could reduce parasite mobility and induced cell death. RNA aptamers (C4 and C5) containing the GUUG motif were isolated, which inhibited *Entamoeba histolytica’s* growth by blocking of EhCFIm25 [102]. Several aptamers developed against parasitic infections are summarized in Table 3.

**Table 3:** A summary of several aptamers developed for the treatment of parasitic infections by SELEX method

Organism	Type of aptamer	Target	Aptamer therapeutic effect	Ref
*E. histolytica*	RNA	Polyadenylation factor EhCFIm25	Inhibited parasite proliferation and led to cell death	[102]
*L. infantum*	DNA	rLiPABP	Affecting the physiological role of PABP	[101]
*P. falciparum*	RNA	EMP1	Disruption and sequestration of parasites Reduced rosette formation	[99]
*T. cruzi*	RNA	Cell adhesion	Inhibit cell invasion	[98]
*T. brucei*	RNA	VSG proteins	Directing antibodies to the surface of the parasite	[103]

*rLiPABP* recombinant 6xHIS-LiPABP protein, *EMP1* erythrocyte membrane protein 1, *VSG* variant surface glycoproteins

### Conclusions and perspectives

Despite significant advances in the treatment of infectious diseases over the past half century, there are still many problems associated with the development of effective therapies and overcoming the side effects of antibiotics and pathogen drug resistance. Aptamers could provide a strong tool for the expansion of new therapeutic factors with the ability to block the function of pathogen microorganisms, also, they are simple and cheap therapeutic agents with less side effects compared to traditional antibiotics. Different therapeutic approaches of aptamers application have been reported in the field of microbial infections. For example, aptamer could inhibit biofilm formation or inhibit the functionality of microbial toxins, therefore, they could be used as new therapeutic tools to combat chronic infections. In addition, aptamers could be conjugated with nanoparticles, siRNA/miRNA, therapeutic drugs, and PDT agents to formulate a new therapeutic composite, which specifically kill bacterial cells. Application of aptamer technology in the treatment of microbial infections is still in its primal phase, and various challenges need to be overcome. This review provides recently developed aptamer-based technologies for microbial infections that illustrated this promising technology as an actual alternative to the traditional approaches in infection therapy. Aptamers are applicable tools in the detection and defense against unknown pathogens (bacterial, fungal, and viral agents) and can become a valuable anti-infective therapeutic tool for clinicians in recent future.

## Data Availability

NA
